# Heart Wall Is Thicker on Postmortem Computed Tomography Than on Ante Mortem Computed Tomography: The First Longitudinal Study

**DOI:** 10.1371/journal.pone.0076026

**Published:** 2013-09-27

**Authors:** Hidemi Okuma, Wataru Gonoi, Masanori Ishida, Yukako Shintani, Yutaka Takazawa, Masashi Fukayama, Kuni Ohtomo

**Affiliations:** 1 Department of Radiology, Graduate School of Medicine, the University of Tokyo, Tokyo, Japan; 2 Department of Radiology, Mutual Aid Association for Tokyo Metropolitan Teachers and Officials, Sanraku Hospital, Tokyo, Japan; 3 Department of Pathology, Graduate School of Medicine, the University of Tokyo, Tokyo, Japan; Institute of Automation, Chinese Academy of Sciences, China

## Abstract

**Objective:**

To evaluate the postmortem changes of the heart wall on postmortem (PM) computed tomography (CT) in comparison with those on ante mortem CT (AMCT), and in comparison with the pathological findings, obtained in the same patients.

**Materials and Methods:**

We studied 57 consecutive patients who had undergone AMCT, PMCT, and pathological autopsy in our tertiary care hospital between April 2009 and December 2010. PMCT was performed within 20 hours after death, followed by pathological autopsy. The cardiac chambers were measured at five sites on both AMCT and PMCT by two board-certified radiologists who were not provided with clinical information. The differences in heart wall thickness between AMCT with and without contrast medium, between AMCT and PMCT, and between PMCT and pathological anatomy were evaluated statistically. Confounding factors of postmortem change such as gender, presence of arteriosclerosis, the organ related to cause of death, age, and elapsed time since death were examined statistically.

**Results:**

No significant differences were observed on AMCT in comparison of contrasted and non-contrasted images. The heart wall was significantly thicker on PMCT than on AMCT (p < 0.0001) at all five measurement sites. The heart wall was significantly thicker on PMCT than on pathology specimens when measured in accordance with pathological standard mensuration. However, no significant difference was observed between PMCT measurements and those of pathology specimens at any site when the papillary muscles and epicardial fat were included. No significant association was found between postmortem change in heart wall thickness and gender, presence of arteriosclerosis, the organ related to cause of death, age, or elapsed time since death.

**Conclusion:**

This is the first longitudinal study to confirm greater thickness of heart wall on postmortem images compared with ante mortem images, in the same patients. Furthermore, the postmortem changes on CT were supported by the pathological findings.

## Introduction

High-resolution imaging modalities such as computed tomography (CT) and magnetic resonance imaging (MRI) are beginning to gain a role in postmortem investigations as adjuncts to more traditional methods in forensic medicine [1–5]. The postmortem CT (PMCT) findings of organs such as the brain, lung, heart, and liver have been described [6–12]. To our knowledge, however, the features of cardiac hypertrophy on PMCT have not been reported. Our aim was to investigate wall thickening of the heart on PMCT in comparison with the ante mortem CT (AMCT) findings, and in comparison with the pathological findings, in cases of non-traumatic in-hospital death. The results revealed significant difference in the thickness of the heart wall between PMCT and AMCT, but no significant difference between PMCT and pathological measurements.

## Materials and Methods

### 1. Study Group

The Research Ethics Committee of the University of Tokyo Hospital approved this study, which was conducted in accord with the principles of the Declaration of Helsinki. Written informed consent was obtained from the next of kin of the donor for all corresponding clinical, pathological, and radiographic data to be used in the study. A total of 97 patients who died non-traumatically in our academic tertiary-care hospital and who underwent AMCT, PMCT, and pathological autopsy between April 2009 and December 2010 were retrospectively enrolled in this study. Exclusion criteria were as follows: (a) age < 20 years; (b) cardiopulmonary resuscitation (CPR) performed; and (c) congenital heart disease, chronic heart failure, cardiomyopathy, cardiac hypertrophy, heart amyloidosis, or previous cardiovascular surgery. The final study population consisted of 57 adult human cadavers (40 male, 17 female); mean age at death was 66 years (range, 21–92 years; median, 71 years). All cadavers were placed in the supine position at room temperature from the time of death until PMCT examination. PMCT was performed at 81–1187 min (median 328 min) after death. In addition to these 57 cases, we also performed AMCT, PMCT, and pathological autopsy on 3 cadavers with dilated cardiomyopathy (2 male, 1 female; 22, 53, and 57 years at death).

### 2. AMCT Imaging Technique

All AMCT studies were performed on 64-detector-row helical CT scanners (Aquilion 64, Toshiba Medical Systems Corporation, Ohtawara, Japan; Discovery CT750 HD and LightSpeed VCT, GE Healthcare, Buckinghamshire, UK) in the craniocaudal direction with the patient in the supine position with arms raised. The scan parameters were as follows: slice thickness, 5 mm; slice interval, 5 mm; rotation time, 0.5 s; and tube voltage, 120 kVp. Tube current was controlled automatically using Volume EC and Auto mA. Image reconstruction was performed at 0.5-mm intervals with a 350-mm field of view and a 512 × 512 image matrix.

### 3. PMCT Imaging Technique

All PMCT studies were performed without contrast medium on a 4-detector-row CT scanner (Robusto, Hitachi Medical Corporation, Tokyo, Japan) in the helical mode in the craniocaudal direction; the cadaver was laid in the supine position with arms placed on either side. The scan parameters were as follows: slice thickness, 2.5 mm; slice interval, 1.25 mm; rotation time, 0.5 s; tube voltage, 120 kVp; and tube current, 250 mA. Image reconstruction was performed at 1.25-mm intervals with a 350-mm field of view and a 512 × 512 image matrix.

### 4. Image Interpretation

**Figure 1:**
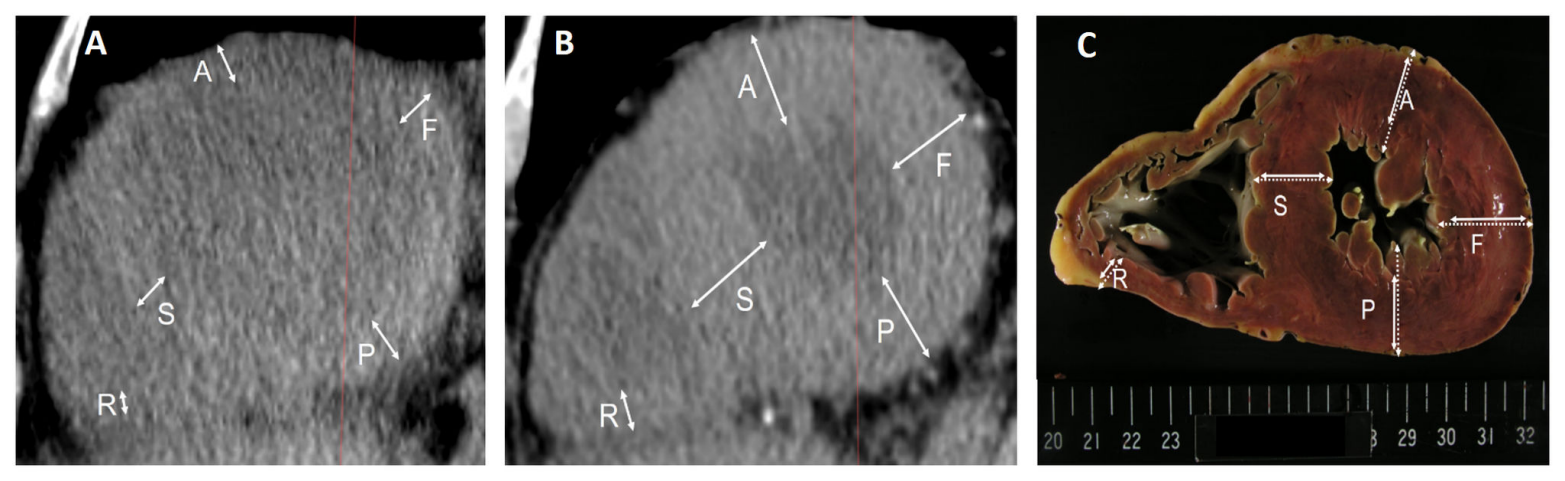
Ante mortem and postmortem measurements of the heart of a 60-year-old man. (a) Multiplanar reconstruction image of ante mortem CT. (b) Multiplanar reconstruction image of postmortem CT. (c) Photograph of the pathological cross-sectional slice. All three of these images represent the same plane. Solid lines represent measurements without including papillary muscles or epicardial fat, according to conventional methods. Dotted lines represent measurements with papillary muscles and epicardial fat. A, anterior wall of the left ventricle; F, left ventricular free wall; P, posterior wall of the left ventricle; S, ventricular septum; R, right ventricular wall.

CT images were reviewed on a three-dimensional (3D) workstation (ZioTerm2009; Ziosoft, Inc., Tokyo, Japan) to obtain multiplanar reconstruction (MPR) images vertical to the long axis of the left ventricle, transecting the apex and the center of the mitral valve. A slice at approximately one third of the distance from the apex, corresponding to the pathological section of the heart, was chosen for analysis. Using the MPR images, we measured the cardiac chambers at five sites: anterior wall of the left ventricle, left ventricular free wall, posterior wall of the left ventricle, ventricular septum, and right ventricular wall (Figure 1a-b). Image analysis was performed by two board-certified radiologists who were not provided with clinical information. The best fit was determined by agreement between the radiologists. The postmortem and most recent ante mortem chest CT images were compared.

### 5. Pathological Analysis

Hearts were studied before formalin fixation on photographs printed with standard scale. Each heart was cross-sectioned into slices approximately 1 cm thick, and a slice at proximately one third of the distance from the apex was chosen for measurement. A surgical pathologist measured myocardial thickness of the anterior wall of the left ventricle, left ventricular free wall, posterior wall of the left ventricle, ventricular septum, and right ventricular wall according to conventional methods [13] at the minimum diameter of each site, excluding papillary muscles and epicardial fat (Figure 1c). Additional measurements including the papillary muscles and epicardial fat were made at each site for further analysis. The PMCT and pathological slices were compared. Slices were determined to be positive for the presence of arteriosclerosis when coronary artery stenosis or atherosclerosis was found pathologically (positive, n = 34; negative, n = 23). The most strongly related cause of death (such as heart failure, respiratory failure, liver failure, loss of blood, or other) was decided based on the clinical course just before death and the pathological findings (heart failure, n = 7; respiratory failure, n = 32; liver failure, n = 4; blood loss, n = 2; other, n = 12). The heart was defined as the organ most related to the cause of death in the case of heart failure; cause of death was classified as ‘other’ in the case of respiratory failure, liver failure, loss of blood, or other causes.

### 6. Statistical Analyses

The average values of the two raters were used for analysis. First we compared the measured values on AMCT with and without contrast medium by paired t-test, to assess whether we could combine the contrasted and non-contrasted AMCT data in further analysis. We then investigated differences between the measured values on AMCT and PMCT, as well as between those on PMCT and pathological measurements, by paired t-test. Postmortem change in heart wall thickness was defined in two ways: as the difference in heart wall thickness between PMCT and AMCT, and as the ratio of heart wall thickness on PMCT to that on AMCT. We analyzed postmortem change in heart wall thickness by unpaired t-test with the following three postmortem changes as confounding factors: gender, presence or absence of arteriosclerosis, and whether or not the heart was the organ most strongly related to the cause of death. Postmortem change in heart wall thickness was also analyzed by linear least squares regression with age and elapsed time since death as additional confounding factors. The level of statistical significance was set at 0.05. Family-wise error was corrected by Bonferroni’s correction for each section. All statistical computing was performed using the free software R, version 3.0 (The R Foundation for Statistical Computing, Vienna, Austria, http://www.r-project.org/).

## Results

The measured values for heart wall thickness on AMCT with and without contrast medium are summarized in Table 1. No significant differences were observed between AMCT with and without contrast medium at any site: anterior wall of the left ventricle, left ventricular free wall, posterior wall of the left ventricle, ventricular septum, or right ventricular wall. We thus combined the contrasted and non-contrasted AMCT data for further analysis: non-contrasted AMCT was used when it was available; otherwise, contrasted AMCT was used.

**Table 1:** Heart wall thickness on AMCT with and without contrast medium.

	Contrasted	Non-contrasted	p value
Anterior wall of the left ventricle (mm; mean ± SD)	10.0 ± 2.9	11.0 ± 1.6	0.25
Left ventricular free wall (mm; mean ± SD)	12.6 ± 3.1	11.0 ± 1.6	0.06
Posterior wall of the left ventricle (mm; mean ± SD)	13.3 ± 3.6	10.9 ± 1.8	0.04^#^
Ventricular septum (mm; mean ± SD)	11.1 ± 3.0	11.2 ± 1.7	0.86
Right ventricular wall (mm; mean ± SD)	4.0 ± 1.4	4.8 ± 0.9	0.13

Statistical analyses were performed by paired t-test. SD, standard deviation; AMCT, antemortem computed tomography ^#^Statistically insignificant when family-wise error was corrected by Bonferroni’s correction

The measured values for heart wall thickness on AMCT and PMCT are summarized in Table 2. The heart wall was significantly thicker on PMCT than on AMCT, at all sites.

**Table 2:** Heart wall thickness on AMCT and PMCT.

	AMCT	PMCT	p value
Anterior wall of the left ventricle (mm; mean ± SD)	10.8 ± 2.8	17.1 ± 3.3	< 0.0001*
Left ventricular free wall (mm; mean ± SD)	12.1 ± 2.9	19.3 ± 3.5	< 0.0001*
Posterior wall of the left ventricle (mm; mean ± SD)	11.7 ± 2.8	18.2 ± 3.2	< 0.0001*
Ventricular septum (mm; mean ± SD)	11.5 ± 2.8	17.3 ± 3.6	< 0.0001*
Right ventricular wall (mm; mean ± SD)	4.7 ± 2.0	7.5 ± 2.6	< 0.0001*

Statistical analyses were performed by paired t-test. SD, standard deviation; AMCT, antemortem computed tomography; PMCT, postmortem computed tomography *Statistically significant

Pathologically measured values for heart wall thickness are summarized in Table 3. The heart wall was significantly thicker on PMCT than on pathology specimens when measured without papillary muscles or epicardial fat according to conventional methods. However, no significant differences were observed between measurements on PMCT and pathology specimens at any site when measurements included papillary muscles and epicardial fat.

**Table 3:** Heart wall thickness on pathology specimens measured with and without papillary muscles and epicardial fat.

	PMCT	Pathology(-)	p value1	Pathology(+)	p value2
Anterior wall of the left ventricle (mm; mean ± SD)	17.1 ± 3.3	12.8 ± 3.0	< 0.0001*	19.2 ± 5.2	0.02^#^
Left ventricular free wall (mm; mean ± SD)	19.3 ± 3.5	13.9 ± 3.0	< 0.0001*	20.5 ± 3.8	0.06
Posterior wall of the left ventricle (mm; mean ± SD)	18.2 ± 3.2	12.5 ± 2.8	< 0.0001*	19.3 ± 3.4	0.10
Ventricular septum (mm; mean ± SD)	17.3 ± 3.6	12.7 ± 3.0	< 0.0001*	17.8 ± 3.8	0.38
Right ventricular wall (mm; mean ± SD)	7.5 ± 2.6	4.1 ± 1.1	< 0.0001*	7.3 ± 1.1	0.61

Statistical analyses were performed by paired t-test. p value^1^, between PMCT and pathological specimen, without papillary muscles or epicardial fat p value^2^, between PMCT and pathological specimen, with papillary muscles and epicardial fat SD, standard deviation; PMCT, postmortem computed tomography; pathology(–), without papillary muscles and epicardial fat; pathology(+), with papillary muscles and epicardial fat *Statistically significant ^#^Statistically insignificant when family-wise error was corrected by Bonferroni’s correction

Table 4, Table 5, and Table 6 summarize the association of postmortem change in heart wall thickness with each of gender, presence of arteriosclerosis, and the organ related to the cause of death. Gender, presence of arteriosclerosis, and heart failure as the cause of death showed no statistically significant association with postmortem change in heart wall thickness.

**Table 4:** Association between postmortem change in heart wall thickness and gender.

	M(PM–AM)	Fe(PM–AM)	p value	M(PM/AM)	Fe(PM/AM)	p value
A (mm; mean ± SD)	6.9 ± 3.8	5.0 ± 4.5	0.12	1.7 ± 0.5	1.6 ± 0.6	0.59
F (mm; mean ± SD)	7.2 ± 4.5	7.2 ± 4.0	0.98	1.6 ± 0.4	1.7 ± 0.4	0.54
P (mm; mean ± SD)	6.9 ± 3.5	5.7 ± 4.0	0.27	1.6 ± 0.4	1.6 ± 0.5	0.75
S (mm; mean ± SD)	6.4 ± 3.9	4.2 ± 3.7	0.05	1.6 ± 0.5	1.4 ± 0.4	0.11
R (mm; mean ± SD)	3.0 ± 2.3	2.2 ± 1.8	0.19	1.8 ± 0.7	1.6 ± 0.5	0.25

Statistical analyses were performed by unpaired t-test. M, male; Fe, female; PM–AM, difference in heart wall thickness between postmortem computed tomography and antemortem computed tomography; PM/AM, ratio of heart wall thickness on postmortem computed tomography to that on antemortem computed tomography; SD, standard deviation; A, anterior wall of the left ventricle; F, left ventricular free wall; P, posterior wall of the left ventricle; S, ventricular septum; R, right ventricular wall

**Table 5:** Association between postmortem change in heart wall thickness and presence of arteriosclerosis.

	(+) (PM–AM)	(-) (PM–AM)	p value	(+) (PM/AM)	(-) (PM/AM)	p value
A (mm; mean ± SD)	6.4 ± 4.6	6.2 ± 3.3	0.87	1.7 ± 0.6	1.7 ± 0.4	0.75
F (mm; mean ± SD)	7.2 ± 4.8	7.2 ± 3.4	0.98	1.7 ± 0.5	1.7 ± 0.4	0.86
P (mm; mean ± SD)	6.5 ± 4.2	6.6 ± 2.8	0.92	1.6 ± 0.4	1.7 ± 0.4	0.64
S (mm; mean ± SD)	5.8 ± 4.2	5.8 ± 3.8	0.96	1.5 ± 0.4	1.6 ± 0.5	0.59
R (mm; mean ± SD)	2.5 ± 2.1	3.3 ± 2.2	0.19	1.6 ± 0.5	1.9 ± 0.8	0.12

Statistical analyses were performed by unpaired t-test. (+), with arteriosclerosis; (–), without arteriosclerosis; PM–AM, difference in heart wall thickness between postmortem computed tomography and antemortem computed tomography; PM/AM, ratio of heart wall thickness on postmortem computed tomography to that on antemortem computed tomography; SD, standard deviation; A, anterior wall of the left ventricle; F, left ventricular free wall; P, posterior wall of the left ventricle; S, ventricular septum; R, right ventricular wall

**Table 6:** Association between postmortem change in heart wall thickness and cause of death.

	H(PM–AM)	O(PM–AM)	p value	H(PM/AM)	O(PM/AM)	p value
A (mm; mean ± SD)	5.9 ± 5.9	6.5 ± 3.8	0.38	1.5 ± 0.5	1.8 ± 0.5	0.28
F (mm; mean ± SD)	5.8 ± 6.7	7.4 ± 3.9	0.35	1.6 ± 0.6	1.7 ± 0.4	0.45
P (mm; mean ± SD)	5.6 ± 6.3	6.7 ± 3.3	0.50	1.5 ± 0.5	1.6 ± 0.4	0.29
S (mm; mean ± SD)	1.6 ± 0.5	5.8 ± 3.9	0.93	1.5 ± 0.4	1.6 ± 0.5	0.62
R (mm; mean ± SD)	1.9 ± 2.0	3.0 ± 2.2	0.24	1.4 ± 0.5	1.8 ± 0.6	0.16

Statistical analyses were performed by unpaired t-test. H, died of heart failure; O, died of other than heart failure; PM–AM, difference in heart wall thickness between postmortem computed tomography and antemortem computed tomography; PM/AM, ratio of heart wall thickness on postmortem computed tomography to that on antemortem computed tomography; SD, standard deviation; A, anterior wall of the left ventricle; F, left ventricular free wall; P, posterior wall of the left ventricle; S, ventricular septum; R, right ventricular wall

Table 7 and Table 8 show the results of correlation analysis for postmortem change in heart wall thickness with each of age and elapsed time since death, revealing no statistically significant correlation.

**Table 7:** Correlation of association between postmortem change in heart wall thickness with age.

	PM–AM	PM/AM
Anterior wall of the left ventricle	0.064	0.090
Left ventricular free wall	0.061	0.063
Posterior wall of the left ventricle	0.060	0.072
Ventricular septum	0.20	0.22
Right ventricular wall	0.086	0.060

Statistical analyses were performed by linear least squares regression. PM–AM, difference in heart wall thickness between postmortem computed tomography and antemortem computed tomography; PM/AM, ratio of heart wall thickness on postmortem computed tomography to that on antemortem computed tomography

**Table 8:** Correlation of association between postmortem change in heart wall thickness with elapsed time since death.

	PM–AM	PM/AM
Anterior wall of the left ventricle	0.21	0.18
Left ventricular free wall	0.11	0.11
Posterior wall of the left ventricle	0.10	0.067
Ventricular septum	0.16	0.10
Right ventricular wall	0.15	0.04

Statistical analyses were performed by linear least squares regression. PM–AM, difference in heart wall thickness between postmortem computed tomography and antemortem computed tomography; PM/AM, ratio of heart wall thickness on postmortem computed tomography to that on antemortem computed tomography

Table 9 and Table 10 summarize postmortem change in heart wall thickness in the cases of dilated cardiomyopathy and in the controls. There was less postmortem change in heart wall thickness in cases of dilated cardiomyopathy compared with control cases.

**Table 9:** Heart wall thickness on AMCT and PMCT in dilated cardiomyopathy.

	AMCT	PMCT
Anterior wall of the left ventricle (mm; mean ± SD)	14.3 ± 1.9	15.7 ± 2.9
Left ventricular free wall (mm; mean ± SD)	12.8 ± 1.4	14.3 ± 1.3
Posterior wall of the left ventricle (mm; mean ± SD)	13.4 ± 2.4	14.8 ± 2.4
Ventricular septum (mm; mean ± SD)	12.6 ± 3.3	16.4 ± 1.5
Right ventricular wall (mm; mean ± SD)	5.4 ± 0.6	%1.1 ± 1.8

SD, standard deviation; AMCT, ante mortem computed tomography; PMCT, postmortem computed tomography

**Table 10:** Postmortem change in heart wall thickness in cases of dilated cardiomyopathy and in controls.

	DC(PM–AM)	C(PM–AM)	DC(PM/AM)	C(PM/AM)
Anterior wall of the left ventricle (mm; mean ± SD)	1.4 ± 4.6	6.5 ± 0.8	1.1 ± 0.3	1.6 ± 1.3
Left ventricular free wall (mm; mean ± SD)	1.5 ± 2.7	7.3 ± 0.6	1.1 ± 0.2	1.6 ± 1.1
Posterior wall of the left ventricle (mm; mean ± SD)	2.8 ± 3.4	6.6 ± 0.0	1.2 ± 0.3	1.6 ± 1.0
Ventricular septum (mm; mean ± SD)	3.8 ± 4.0	6.1 ± 1.2	1.4 ± 0.4	1.5 ± 1.4
Right ventricular wall (mm; mean ± SD)	2.1 ± 1.4	2.8 ± 0.5	1.4 ± 0.2	1.6 ± 1.2

SD, standard deviation; PM–AM, difference in heart wall thickness between postmortem computed tomography and antemortem computed tomography; PM/AM, ratio of heart wall thickness on postmortem computed tomography to that on antemortem computed tomography; DC, dilated cardiomyopathy; C, control

## Discussion

Because of the obvious differences in appearance between contrast-enhanced and plain images, we evaluated whether the measured values of heart wall thickness were equivalent between these two sets of images. No significant difference was found, which enabled us to use either contrasted or non-contrasted images to obtain measurements.

While several previous studies have evaluated the postmortem changes of the cardiovascular system [14–17], few studies have focused on heart wall thickness. Hutchins et al. [18] measured ventricular wall thickness on stereoscopic radiographs of hearts obtained at autopsy, and concluded that the left ventricular free wall is the thickest, followed by the interventricular septum; the right ventricular free wall is the thinnest. The present results are generally in agreement with theirs.

In the present study, the heart wall was significantly thicker on PMCT than on AMCT in the same patients. Lewy et al. [19] reported that PMCT showed no specific findings for rigor mortis, and that rigor did not affect the CT attenuation, size, or shape of skeletal muscles. However, it is well known in forensic medicine and pathology that rigor mortis causes contraction of the heart, which manifests as a hypertrophic appearance of the ventricular walls [20–22]. Postmortem change in heart wall thickness should be associated with elapsed time since death if rigor mortis principally accounts for heart wall thickness on PMCT, because the presence and degree of rigor mortis generally changes in the period between 1–2 hours and 24–36 hours after death [23]. All PMCT scanning in the present study was performed between 1 and 20 hours post mortem, which coincides with the timing of rigor mortis. Our results showed that the heart wall becomes thicker post mortem, which is consistent with the knowledge in forensic medicine and pathology that the ventricular walls appear hypertrophic after death. However, because the present study revealed few associations between postmortem change in heart wall thickness and elapsed time after death, it is possible that this change may occur within the first few hours or even minutes after death, which is earlier than the observation time of the present study.

We also investigated possible confounding factors of postmortem change such as gender, presence of arteriosclerosis, the organ most strongly related to cause of death, and age. There was little association between these factors and postmortem change in heart wall thickness, which indicates that change in heart wall thickness is a general postmortem finding regardless of these factors.

Dilated cardiomyopathy is characterized by ventricular chamber enlargement and systolic dysfunction with normal left ventricular wall thickness [24]. The histologic features of dilated cardiomyopathy are nonspecific, and pathological findings range from minimal change in myocyte size to typical features of myofiber loss, interstitial fibrosis, and marked change in myofiber size [25]. It has been clarified that dilated cardiomyopathy is partly caused by mutations in the genes that encode for proteins of the myocyte contractile apparatus, the myocyte cytoskeleton, and nuclear envelope, as well as proteins involved in calcium homeostasis [26]. Because the myocardium is injured and degenerates to various extents in dilated cardiomyopathy, we would expect to observe reduced postmortem change in heart wall thickness in these cases compared with controls, which is confirmed by the results of the present study. Furthermore, this leads to the supposition that ante mortem myocardial volume or thickness is positively correlated to postmortem change in heart wall thickness, although no correlation was found for confounding factors of postmortem change such as gender, presence of arteriosclerosis, the organ related to the cause of death, and age.

The heart wall was significantly thicker on PMCT than on pathology specimens when measured by conventional mensuration, which did not include papillary muscles or epicardial fat. However, no significant differences were observed between measurements on PMCT and pathology specimens when measurements included papillary muscles and epicardial fat. This could be because the resolution of the CT images was not high enough to enable distinction of papillary muscles and epicardial fat from myocardia, and because heart wall measurements on PMCT had included these elements.

Cardiac motion artifact is a major concern on AMCT that clearly does not arise on PMCT. Because we did not use electrocardiographic triggering on AMCT, these images were a composite of systolic and diastolic phases. According to the duration of each phase, it is assumed that on AMCT the systolic phase comprised approximately 0.4 of the entire cardiac cycle, while the other 0.6 was diastolic phase [27]. In contrast, the heart outlines on PMCT are considered to be close to those during the diastolic phase, because mean circulatory pressure of deceased subjects (approximately 7 mmHg) is similar to the end-diastolic pressure of the right or left ventricle of living subjects[14]. . The heart wall becomes 40% to 60% thicker in the systolic phase compared with the diastolic phase [28], while in the present study, it was approximately 60% thicker on PMCT compared with AMCT. If there were no postmortem changes, then heart wall thickness should have been noticeably thinner on PMCT than on AMCT, which is the opposite to what was shown in the present results.

Our study has another limitation. We did not consider underlying disease, preservation of cadavers, atmospheric temperature, or humidity, among other factors, which do affect postmortem changes [29,30]. However, we consider that these factors would have had little effect on our conclusion, because we excluded heart disease from analysis and all the cadavers were preserved under automatically regulated conditions at our hospital.

## Conclusions

This is the first longitudinal study to elucidate that the heart wall is significantly thicker on PMCT than on AMCT in cases of non-traumatic in-hospital death. Furthermore, the postmortem changes on CT were supported by the pathological findings.
