# Correction: Heart Wall Is Thicker on Postmortem Computed Tomography Than on Ante Mortem Computed Tomography: The First Longitudinal Study

**DOI:** 10.1371/annotation/7bae7ece-15f5-43ca-9f98-592df8543978

**Published:** 2013-10-09

**Authors:** Hidemi Okuma, Wataru Gonoi, Masanori Ishida, Yukako Shintani, Yutaka Takazawa, Masashi Fukayama, Kuni Ohtomo

In the article title, the word "antemortem" was misspelled. The correct title is: "Heart Wall Is Thicker on Postmortem Computed Tomography Than on Antemortem Computed Tomography: The First Longitudinal Study." The correct citation is: Okuma H, Gonoi W, Ishida M, Shintani Y, Takazawa Y, et al. (2013) Heart Wall Is Thicker on Postmortem Computed Tomography Than on Antemortem Computed Tomography: The First Longitudinal Study. PLoS ONE 8(9): e76026. doi:10.1371/journal.pone.0076026.

In addition, there were multiple errors in the Author Contributions Statement. The Author Contributions Statement should read: "Conceived and designed the experiments: HO WG. Performed the experiments: WG MI YS. Analyzed the data: HO WG. Contributed reagents/materials/analysis tools: YT MF. Wrote the manuscript: HO WG KO." 

